# Breeding innovations focused on drought tolerance, and Nitrogen and phosphorus uptake, for sustainable and climate-resilient sugarcane

**DOI:** 10.3389/fpls.2026.1925822

**Published:** 2026-10-05

**Authors:** María Francisca Perera, Josefina Racedo, Aline Cestaro Salla Sá, Joao Vitor de Mello Vomero, Thiago Willian Almeida Balsalobre, Marcio Roberto Soares, Monalisa Sampaio Carneiro

**Affiliations:** 1 Instituto de Tecnología Agroindustrial del Noroeste Argentino (ITANOA), Estación Experimental Agroindustrial Obispo Colombres (EEAOC) – Consejo Nacional de Investigaciones Científicas y Técnicas (CONICET), Las Talitas, Tucumán, Argentina; 2 Departamento de Biotecnologia e Producão Vegetal e Animal, Centro de Ciências Agrarias, Universidade Federal de São Carlos (UFSCar), Araras, São Paulo, Brazil; 3 Sugarcane Breeding Program of Rede Interuniversitária para o Desenvolvimento do Setor Sucroenergético (RIDESA)/UFSCar, Araras, São Paulo, Brazil; 4 Departamento de Recursos Naturais e Proteção Ambiental, Centro de Ciências Agrarias, Universidade Federal de São Carlos (UFSCar), Araras, São Paulo, Brazil

**Keywords:** drought tolerance, NUE, phosphorus uptake, *Saccharum* spp., sustainability

## Abstract

Sugarcane (*Saccharum* spp.) is a strategic agro-industrial crop with a central role in global sugar production, bioethanol generation, and the development of sustainable bioeconomy systems. Beyond sugar, sugarcane by-products such as bagasse, molasses, and vinasse represent valuable resources for renewable energy, biofuels, biopolymers, and circular production systems. However, climate change threatens sugarcane productivity worldwide through increased temperatures, irregular rainfall patterns, and intensified drought events, directly affecting biomass accumulation, photosynthesis, and crop yield. This review summarizes current advances in climate-resilient sugarcane breeding and sustainable nutrient management strategies, with emphasis on drought tolerance, nitrogen-use efficiency (NUE), and phosphorus-use efficiency (PUE). Drought induces significant physiological, biochemical, and molecular alterations in sugarcane, including reduced growth, impaired photosynthetic activity, osmotic imbalance, and oxidative stress. Advances in breeding programs, omics technologies, molecular markers, and genetic engineering have improved the understanding of drought tolerance mechanisms and enabled the identification of stress-responsive genes, transcription factors, and candidate molecular markers associated with resilience. In parallel, improving NUE and PUE has become essential to reduce fertilizer dependence and mitigate environmental impacts associated with nutrient losses. Integrated management strategies involving enhanced-efficiency fertilizers, precision agriculture, biological Nitrogen fixation, microbial inoculants, residue recycling, and the development of nutrient-efficient genotypes have shown promising results. Although significant progress has been achieved in genomics and biotechnology, the genetic complexity of sugarcane and limited knowledge of nutrient-related pathways still constrain the development of highly efficient cultivars. The integration of advanced breeding technologies with sustainable agronomic practices will be crucial to ensure resilient and environmentally sustainable sugarcane production under future climate scenarios.

## 1 Introduction

Sugarcane (*Saccharum* spp.) is one of the most versatile and strategically important crops worldwide (Wannaree, 2026). Approximately 80% of the world’s sugar and a substantial share of renewable bioethanol are derived from sugarcane, and its exceptional biomass productivity, high photosynthetic efficiency have made it a key feedstock for integrated biorefineries. Industrial by-products such as bagasse, straw, molasses, filter cake, and vinasse increasingly valorized in circular production systems, generating bioenergy, biochemicals and biofertilizers (Deuber et al., 2023). In addition, these by-products have also shown potential as feedstocks for sustainable aviation fuels through hydrothermal liquefaction, contributing to decarbonization strategies (Deuber et al., 2023). This diversification has positioned sugarcane as a cornerstone of the low-carbon bioeconomy, supporting renewable energy production, greenhouse gas mitigation, and sustainable industrial development (de Matos Nogueira, 2025).

Despite its remarkable potential, sugarcane production is increasingly threatened by climate change, including rising temperatures and irregular precipitation patterns (Zhou et al., 2025; IPCC, 2023; **Table 1**). These environmental changes induce physiological alterations such as reduced biomass accumulation, increased evapotranspiration, altered photosynthetic rates, and impaired vegetative growth, ultimately affecting crop productivity (Dutta and Dutta, 2025). Moreover, extreme environmental conditions can delay tillering, reduce stalk elongation, and impair maturation and sugar accumulation (Verma et al., 2022; Kulrat et al., 2025). Climate change also affects disease cycles, weed dynamics, and pest behavior, increasing pressure on crop productivity (Kumar et al., 2023; Msomba et al., 2024). All the aforementioned emphasizes the need to develop climate-resilient varieties with greater plasticity and capacity to maintain productivity through genetic research and adaptive breeding strategies (Linnenluecke et al., 2020; Khan, 2023; Msomba et al., 2024) by integrating multiple traits. Among the major challenges that need to be addressed for sustainable sugarcane production are drought tolerance, Nitrogen-use efficiency (NUE), and phosphorus-use efficiency (PUE), three highly interconnected traits that collectively determine plant productivity. Water availability strongly influences nutrient acquisition, whereas efficient Nitrogen and phosphorus utilization enhances physiological resilience to drought. Consequently, improving these traits simultaneously represents a key objective for the development of climate-resilient sugarcane cultivars. Drought is a major limiting factor capable of reducing productivity by up to 60% (Srithawong et al., 2026), whereas improving NUE and PUE is essential to reduce fertilizer dependence and environmental impacts. The relevance of these traits is reflected in global sustainability indicators, such as the Environmental Performance Index (EPI; https://epi.yale.edu/measure/2024/EPI), which integrates environmental performance and nutrient management indicators, including the Sustainable Nitrogen Management Index (SNMI) and the Phosphorus Surplus Index (**Table 2**). Differences among major sugarcane-producing countries highlight the need for strategies that combine agronomic practices and genetic improvement through genome sequencing, gene editing, high-throughput phenotyping, and predictive breeding tools (Msomba et al., 2024; Srithawong et al., 2026) to optimize resource use and reduce environmental impacts. Therefore, this review synthesizes current advances in drought tolerance, nitrogen- and phosphorus-use efficiency, and emerging predictive breeding technologies, highlighting future opportunities for the development of productive, sustainable and climate-resilient sugarcane production systems (**Figure 1**).

**Table 1:** Climate change outlook in the main sugarcane producing countries and the projected impact on crop productivity.

Country	% of Global sugar production	Annual production (Mt)	MAT (°C)	MAP (mm yr^−1^)	Projected warming by 2100 (°C)	Dominant system	Climate-induced water scarcity risk	Projected yield change (%)
Brazil	24	650 - 700	22 - 26	1,200 - 1,600	+2.0 to +4.5	Rainfed	Moderate to high	+8 to −20
India	15	~400	24 - 30	900 -1,500	+1.5 to +3.0	Irrigated (monsoon)	High	−10 to −25
China	6	110 -120	20 - 25	1,100 - 1,800	+1.8 to +3.5	Mixed	Moderate	−5 to +10
Thailand	6	90 - 100	26 - 30	1,000 - 1,400	+2.0 to +4.0	Rainfed	High	−10 to −30
Pakistan	3	70 - 80	23 - 29	200 - 600	+2.0 to +3.0	Irrigated (Indus Basin)	Very high	−20 to −40
Australia	2	30 - 35	18 - 24	1,000 - 2,000	+1.5 to +2.5	Mixed	Moderate	+5 to +15
South Africa	1	18 - 20	20 - 25	600 -1,200	+2.0 to +4.0	Irrigated	High	−10 to −25

MAT, minimal annual temperature; MAP, minimal annual precipitation.

**Table 2:** Environmental performance and nutrient management indicators in major sugarcane-producing countries.

Sugarcane production rank	Country	EPI (rank/score)	SNMI score	Phosphorus surplus score
1	Brazil	47/53.0	78.8	30.2
2	India	176/27.6	51.6	39.4
3	Thailand	90/45.7	48.9	60.7
4	China	156/35.4	58.8	30.1
5	Pakistan	179/25.5	33.9	35.3
6	Mexico	97/44.2	48.5	50.1
7	Colombia	63/49.7	47.4	28.8
8	Australia	23/63.1	49.7	51.8
9	Indonesia	163/33.6	52.5	49.3
10	Guatemala	166/32.5	64.7	44.3
11	United States	35/57.2	76.6	80.7
12	Philippines	169/32.1	73.7	86.1
13	South Africa	106/42.7	45.8	55.0
14	Argentina	81/47.0	87.7	100.0

EPI, Environmental Performance Index. Source: https://epi.yale.edu/measure/2024/EPI. SNMI, Sustainable Nitrogen Management Index, a metric that assesses how efficiently countries balance nitrogen fertilizer inputs with crop productivity. Phosphorus Surplus: indicator based on the difference between phosphorus inputs and crop removal, reflecting the potential risk of phosphorus losses to the environment. Higher scores indicate better environmental performance and more sustainable nutrient management practices. Countries are ranked according to sugarcane production data reported by FAOSTAT (EPI, 2024; FAOSTAT crop nutrient budget database).

**Figure 1:**
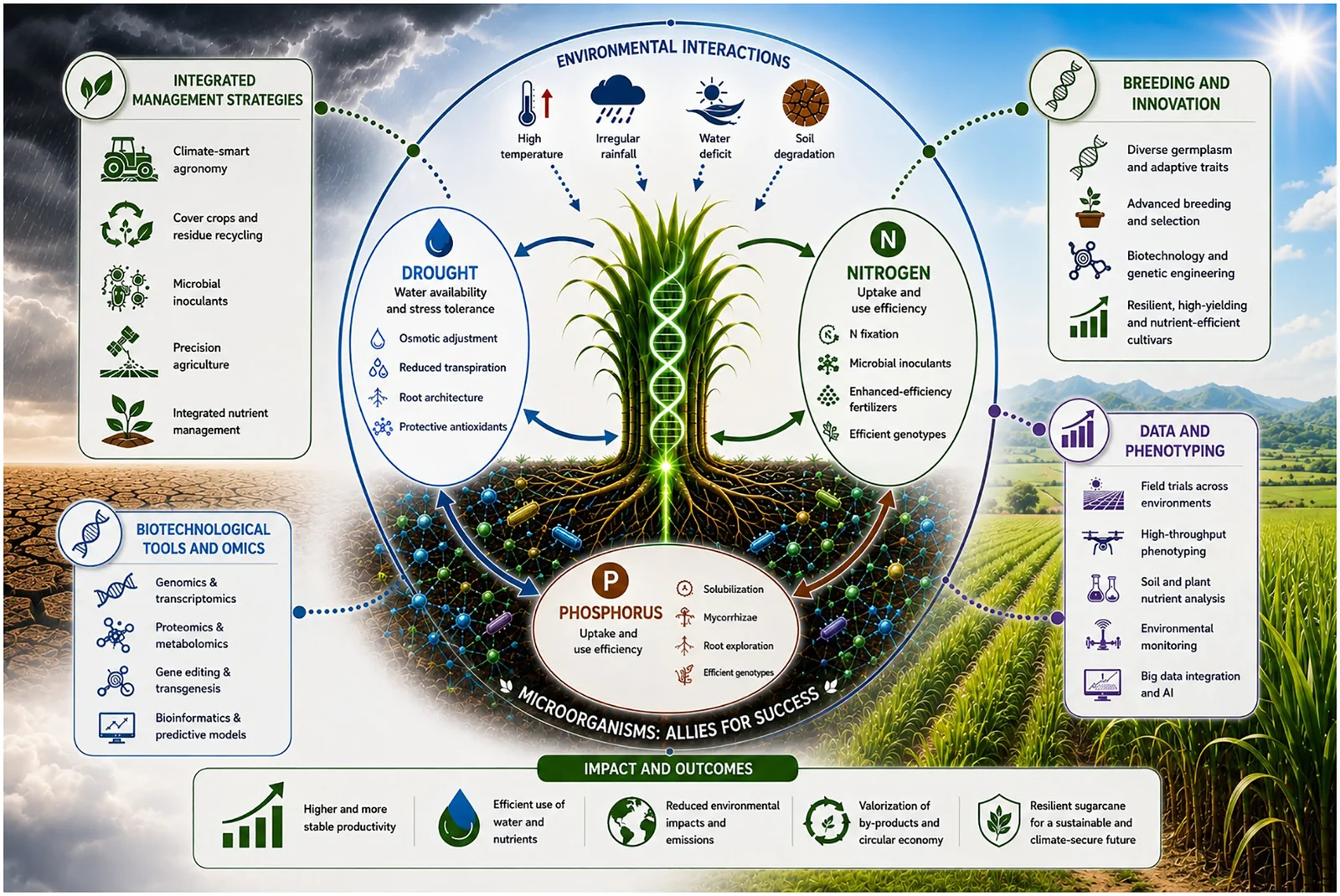
Integrated framework for sustainable and resilient sugarcane. The framework integrates environmental stresses, water and nutrient use efficiency, plant–microorganism interactions, biotechnological and omics tools, breeding, phenotyping, and integrated management strategies to improve productivity, resource-use efficiency, and resilience while reducing environmental impacts.

## 2 Sugarcane drought resilience

Sugarcane exhibits high sensitivity to water deficit throughout its phenological cycle, with three critical periods determining the extent of yield loss: drought at germination and early establishment reduces bud sprouting and stand density (Reyes et al., 2020), water deficit during the tillering and stalk elongation phase most severely compromises biomass accumulation (Robertson et al., 1999; Mahadevaiah et al., 2021), and prolonged drought during the maturation period restricts late-season growth and, depending on timing and duration, can also affect sucrose accumulation (Sales et al., 2015; Ferreira et al., 2017). Drought stress in sugarcane induces leaf wilting, decreases leaf water content and green leaf area, and consequently reduces photosynthetic capacity. These effects impair plant growth, affecting internode length, stem diameter, plant height, and stalk weight, ultimately resulting in yield losses. Under prolonged drought conditions, tillering, functional leaf number, total leaf area, and solar energy capture are also reduced (Mall et al., 2022). At physiological and biochemical levels, drought stress enhances antioxidant enzyme activity, including catalase, superoxide dismutase, and peroxidase, while increasing the accumulation of osmotic adjustment compounds such as soluble sugars and proline. In parallel, drought reduces transpiration rate, stomatal conductance, and chlorophyll content, affecting photosynthetic performance. Consequently, antioxidant enzyme activity, osmotic adjustment capacity, chlorophyll content, and chlorophyll fluorescence parameters have been proposed as useful indicators for evaluating drought tolerance in sugarcane (Li et al., 2026).

### 2.1 Conventional breeding

Climate change is increasing the frequency, intensity, and unpredictability of drought events, making the development of sugarcane cultivars capable of maintaining high productivity and yield stability across contrasting water regimes a major priority for breeding programs (Meena et al., 2020). Consequently, considerable efforts over the past two decades have focused on identifying physiological and morphological traits associated with drought adaptation that could be incorporated into selection schemes. These include stomatal conductance, photosynthetic capacity and efficiency, chlorophyll content (SPAD), transpiration, canopy temperature, water-use efficiency (WUE), intrinsic transpiration efficiency (iTE), root architecture, osmoprotectant accumulation (e.g., proline and trehalose), and leaf morphological characteristics, all of which have been investigated as candidate traits for drought tolerance (Basnayake et al., 2015). While these studies have substantially advanced our understanding of drought adaptation mechanisms, only a limited number of traits have demonstrated sufficient robustness and predictive value for routine application in practical breeding. More recently, research has increasingly shifted toward field-based validation of physiological and agronomic traits as reliable selection criteria for sugarcane breeding programs (Basnayake et al., 2015; Natarajan et al., 2021; Alarmelu et al., 2025). In particular, canopy conductance has been proposed as a robust physiological trait for early selection in sugarcane breeding (Basnayake et al., 2015). Across contrasting environments, early-season canopy conductance showed a strong genetic correlation with final cane yield (0.70–0.90), and this relationship was maintained under water-deficit conditions, reaching values as high as 0.94 in rainfed trials. These results support its use as a reliable indirect selection criterion for the early identification of drought-tolerant, high-yielding genotypes. Similarly, Natarajan et al., 2021 reported that field-measured iTE exhibited high broad-sense heritability (H^2^ = 0.63–0.67) and strong genetic correlations with total cane yield (TCH; rg = 0.72–0.75) under both irrigated and rainfed conditions. However, its usefulness as a selection criterion was maximized within a stomatal conductance (g_s_) range of 0.1–0.4 mol m^−2^ s^−1^, whereas measurements obtained under more severe water stress (g_s_ < 0.1 mol m^−2^ s^−1^) were affected by non-stomatal limitations to photosynthesis, reducing both the heritability and the predictive ability of iTE for yield. Likewise, field evaluations have demonstrated that yield components such as stalk number, stalk height, stalk weight, total biomass, and cane volume can reliably discriminate drought-tolerant genotypes (Alarmelu et al., 2025). Collectively, these studies indicate that sugarcane drought breeding is shifting from the identification of stress-responsive traits toward the validation of robust, heritable, and breeding-relevant phenotypes with demonstrated predictive value for yield, facilitating their integration into modern breeding strategies for the development of climate-resilient cultivars.

Another approach to generating genetic variability for drought tolerance is through EMS-induced mutagenesis. Several studies have identified EMS-derived sugarcane mutants with improved responses to water deficit through *in vitro* screening followed by greenhouse or pot evaluations, which are useful for the initial identification of promising drought-tolerant genotypes (Masoabi et al., 2023). Complementing these controlled-environment studies, field validation is particularly valuable because it allows the assessment of plant performance under the complex combination of environmental factors that occur under agricultural conditions. In this context, Rungmekarat et al. (2026) evaluated 59 EMS-induced mutant clones under field conditions and identified genotypes with superior stalk weight per clump under drought while maintaining sugar content comparable to the parental cultivar. These results provide valuable evidence that EMS-induced mutagenesis can generate genetic variation with potential for improving drought resilience and yield performance under field conditions.

### 2.2 Biological mechanisms of adaptation to drought

Physiological acclimation mechanisms may also contribute to drought resilience in sugarcane. Initial evidence from greenhouse experiments showed that plants previously exposed to cyclic water deficit displayed enhanced performance during subsequent drought episodes, including greater root biomass, higher root-to-shoot ratios, and improved biomass accumulation after rehydration, suggesting the existence of stress memory mechanisms that enhance tolerance to recurrent drought. Because sugarcane is propagated vegetatively, these stress-induced physiological adjustments may also be transmitted to daughter propagules, allowing newly established plants to retain a memory of the parental stress experience (Marcos et al., 2018a, 2018b). Building on these findings, Garcia et al. (2021) demonstrated that a prior drought event induced systemic physiological acclimation, allowing plants to better withstand a second water deficit through smaller reductions in photosynthesis, improved water-use efficiency and photochemical performance, and reduced physiological plasticity, indicating that previous stress exposure lessened the physiological impact of subsequent drought. They further proposed that reduced physiological plasticity under recurrent stress could serve as a useful trait for identifying drought-tolerant genotypes. More recently, these observations were extended to field conditions. Propagules derived from parental plants previously exposed to drought exhibited improved photosynthetic performance, larger root systems, enhanced stalk yield, greater shoot biomass, and superior juice quality, demonstrating that intergenerational drought memory can translate into agronomically relevant improvements under commercial growing conditions. The magnitude of these responses; however, depended on both the genotype and the developmental stage at which the parental plants experienced water deficit (Pissolato et al., 2025).

Plant adaptation to drought also relies on interactions with beneficial rhizosphere microorganisms, which may complement intrinsic physiological responses by enhancing plant performance under water-limited conditions. Rhizosphere microbial communities play an important role in sugarcane adaptation to drought. Drought stress has been associated with shifts in the composition of rhizosphere bacterial communities by enriching different microbial orders positively correlated with more efficient root morphology, likely mediated by changes in soil nutrient availability and enzyme activities. These microbial shifts have also been linked to changes in soil enzymatic activity and plant photosynthesis, suggesting a potential role of the rhizosphere microbiome in enhancing drought adaptation (Liu et al., 2021). In addition, Pereira et al. (2022) showed that the composition and functional potential of the rhizosphere microbiota are modulated by the host genotype under water-limited conditions, highlighting the selective recruitment of bacterial taxa that may contribute to drought adaptation. Together, these studies indicate that drought-induced changes in the sugarcane rhizosphere microbiome are strongly influenced by host genotype, although the specific bacterial taxa associated with drought adaptation may vary among cultivars. Among the bacterial groups associated with these responses, Rhizobiales and Streptomycetales have been consistently highlighted as key taxa with the potential to enhance plant resilience through the production of secondary metabolites, improved nutrient availability, and the promotion of plant growth (Chen et al., 2024).

### 2.3 Breeding innovations

Despite its historical success conventional breeding is still limited by long selection cycles, the difficulty of combining multiple polygenic traits into elite genotypes, and genome complexity. Therefore, molecular and biotechnological approaches have been increasingly implemented to accelerate the development of improved sugarcane varieties (Srithawong et al., 2026).

#### 2.3.1 Candidate gene identification: transcriptomic, proteomic, and metabolomic studies

Recent research focused on genomics, transcriptomics, proteomics, and metabolomics have expanded the understanding of molecular mechanisms underlying drought adaptation (**Table 3**). Among these approaches, transcriptomic analyses have revealed that drought tolerance in sugarcane is regulated by complex gene networks involving stress perception and signal transduction, hormonal signaling, transcriptional regulation, osmotic adjustment, antioxidant defense, and primary and secondary metabolism (Khueychai et al., 2015; Tang et al., 2023; Wang et al., 2025b). Key pathways include abscisic acid (ABA) and ethylene signaling, reactive oxygen species (ROS) scavenging, flavonoid biosynthesis, carbohydrate and energy metabolism, amino acid and lipid metabolism, water transport through aquaporins (expected to support the high WUE), and maintenance of photosynthetic activity. In addition, transcription factors such as *NAC* and *MYB*, together with genes encoding antioxidant enzymes (e.g., APX, SOD, and LEA proteins), protein degradation machinery, and root development regulators, have been consistently associated with enhanced drought tolerance.

**Table 3:** Transcriptomic studies for drought tolerance, NUE and PUE in sugarcane.

Trait	Methodology (reference)	Pathways/genes detected
Drought	cDNA-AFLP (Vantini et al., 2015)	communication pathways and signal transduction, energy, secondary metabolism, growth and development
	Illumina HiScanSQ System and HiSeq 2500 platforms (Belesini et al., 2017)	ascorbate peroxidase, *MYB, E3 SUMO-Protein ligase SIZ2, co-enzyme A ligase* (biosynthesis of flavonoids) and aquaporin
	HT-SuperSAGE (da Silva et al., 2017)	ethylene stress attenuation, root growth, protein degradation, plant proteases, oxidative detoxification, fatty acid synthesis, amino acid transport, and carbohydrate metabolism
	Microarray analysis (Contiliani et al., 2022)	specific genes (*EL3*, *IAA*, *Stellacyanin*, and *GSTU*) were consistently upregulated in tolerant genotypes under natural drought conditions
	RNA-seq (Contiliani et al., 2023)	Calvin cycle and transport of water and carbon dioxide
	Illumina and PacBio (Wang et al., 2025b)	NAC transcription factors, ABA signaling components (*NACA1, ERA1*), and antioxidant defense systems (PER70, SODF1, SODF2, LEA proteins)
NUE	Digital mRNA expression profiling (de Matos Nogueria et al., 2005)	*scGS1.a* and *scGS1.b* (both cytosolic) can be important for Nitrogen assimilation in sugarcane, being responsible for its performance to grow with low Nitrogen input.
	Rolling circle amplification (RCA) technology (Luo et al., 2023)	Overrepresentation of carbohydrate and amino acid metabolism and transmembrane transport genes in one accession, and an enrichment of lipid metabolism and single-organism processes genes in another one, suggesting that divergent metabolic strategies are driving N-related processes.
	RNA-seq (Nong et al., 2023)	During endophytic Nitrogen-fixing bacterial colonization, differentially accumulated metabolites (DAMs) and differentially expressed genes (DEGs) were enriched in the flavonoid biosynthesis pathway. In sugarcane roots, the upregulation of *HCT, CHS*, and *PGT1* was associated with the conversion of p-coumaroyl-CoA into homoeriodictyol chalcone and 5-deoxyleucopelargonidin, suggesting that flavonoid metabolism may contribute to the molecular response of sugarcane to endophytic diazotroph colonization.
	Comparative transcriptome analysis (Hui et al., 2025)	Subgenome analysis highlighted the contribution of *S. spontaneum* to the high NUE of modern sugarcane by revealing genes associated with Nitrogen and carbohydrate metabolism, photosynthesis, and amino acid metabolism. *ScNRT2.3*, which encodes a plasma membrane-localized high-affinity nitrate transporter was identified. Heterologous expression of *ScNRT2.3* enhanced *Arabidopsis* growth under low-nitrate conditions, supporting its potential role in N acquisition under N-limiting conditions. Moreover, interaction between *NAR2.1* and *NRT2.3* was shown to form a conserved protein module involved in the response of sugarcane roots to low-N stress.
	Sequencing of cDNA library (Kiet et al., 2025)	Transcription factor *ScMYB61.1* identified as a promising genetic target for improving NUE and breeding high-NUE cultivars.
	RNA-Seq analysis (Wang et al., 2025a)	Reconstruction of N metabolism and zeatin biosynthesis co-expression networks revealed their coordinated regulatory roles in sugarcane responses to varying N availability. A dynamic and concentration-dependent transcriptional regulatory network in leaves was identified, highlighting candidate genes and regulatory pathways that may contribute to N adaptation.
	RT-qPCR analysis (Qin et al., 2026)	Higher glutamine synthetase (*GS*) and glutamate synthase (*GOGAT*) activities, together with earlier and stronger induction of the high-affinity nitrate transporters *NRT2* and *NRT2.2*, characterized the high-NUE genotype. Network analysis identified NRT2/NRT2.2 as central hubs linking nitrate uptake with *GS/GOGAT*-mediated assimilation. Early activation of nitrate transport promotes downstream N assimilation and sustains growth under N limitation, providing potential molecular targets for breeding high-NUE sugarcane cultivars.
PUE	Genome-Wide In Silico and RNA-Seq (Murugan et al., 2022)	Phosphate acquisition appears to be closely integrated with responses to abiotic stress. Twenty-three *ShPHT* genes were identified belonging to five phosphate transporter subfamilies (PHT1–PHT4 and PHO1), with several members involved in phosphate transport and stress responses. Under salinity stress, *ShPHT1-1, ShPHT1-2*, and *ShPHT1–3* were strongly upregulated in roots, suggesting that regulation of phosphate transport may contribute to the coordination of P acquisition and stress adaptation.

Transcriptomic datasets have also provided valuable resources for molecular breeding. Functional markers, including SSRs and SNPs identified within drought-responsive genes, have potential applications in marker-assisted selection. Likewise, genes consistently upregulated in drought-tolerant genotypes under natural field conditions, such as *Stellacyanin* and *GSTU*, have been proposed as promising molecular markers for drought tolerance screening (Contiliani et al., 2022).

Notably, transcriptome analyses have also uncovered numerous differentially expressed genes encoding proteins of previously unknown function. One of these, *Scdr2* (Sugarcane drought-related 2), has emerged as a promising candidate for improving abiotic stress tolerance. Overexpression of *Scdr2* in transgenic tobacco reduced oxidative damage while increasing photosynthetic activity, stomatal conductance, transpiration, and internal CO_2_ concentration under drought and salinity stress, highlighting its potential as a target for enhancing sugarcane resilience (Begcy et al., 2012, 2019).

Proteomic analyses have furthermore provided complementary insights into the molecular mechanisms underlying drought tolerance by identifying proteins that accumulate in response to water deficit. Several comparative studies between drought-tolerant and drought-sensitive cultivars have consistently shown that tolerance is associated with the differential abundance of proteins involved in photosynthesis, energy and carbon metabolism, signal transduction, antioxidant defense (Jangpromma et al., 2007; Almeida et al., 2013; Budzinski et al., 2019), and cell wall metabolism (Salvato et al., 2019). Several proteins associated with photosynthetic performance, including fructose-bisphosphate aldolase and oxygen-evolving enhancer protein, as well as antioxidant enzymes such as superoxide dismutase (SOD), were found to accumulate at higher levels in tolerant genotypes, contributing to the maintenance of cellular homeostasis under drought conditions (Khueychai et al., 2015). In addition, the drought-inducible protein SoDip22 was identified as an ABA-responsive protein expressed in bundle sheath cells, suggesting an important role for hormone-mediated signaling during drought adaptation (Sugiharto et al., 2002). Together, these findings indicate that proteomic approaches not only validate transcriptomic responses but also identify key proteins and biochemical pathways that contribute directly to drought resilience.

Metabolomic profiling has likewise provided valuable insights into the biochemical responses to drought stress. As example, in a drought-stressed sugarcane variety 166 differentially accumulated metabolites, including amino acids, alkaloids, and lipids were identified. These changes were mainly associated with the enrichment of metabolic pathways involved in phenylalanine metabolism and arginine and proline metabolism, highlighting the importance of osmotic adjustment, antioxidant defense, and stress-related secondary metabolism in drought adaptation (Yang et al., 2023). Wang et al. (2025b) combined transcriptomic and metabolomic profiling of drought-stressed sugarcane, identifying proline and the quinoline derivative 6-methylquinoline as key stress-responsive metabolites, and concluded that application of 6-methylquinoline enhanced drought tolerance with minimal growth penalties. Similarly, network-based integration of proteomic and metabolomic data from contrasting drought resistance cultivars showed that distinct biological processes drive genotype-specific adaptation. Translation-related pathways dominated the tolerant cultivar’s response, whereas stress and energy metabolism were more prominent in the susceptible genotype (Budzinski et al., 2019).

#### 2.3.2 Molecular marker-based approaches: MAS, GWAS, and genomic selection

In parallel, molecular breeding approaches have evolved from low-throughput markers toward genome-wide strategies (**Table 4**). Early studies relied on molecular markers such as RAPD-derived SCARs, SSRs, ISSR and TRAP markers to identify genetic variation associated with drought responses in genetic diversity studies. For example, Srivastava et al. (2012) developed a SCAR marker capable of distinguishing drought-tolerant sugarcane genotypes, demonstrating the potential application of marker-assisted selection (MAS); however, their effective implementation has not been demonstrated. High-throughput genotyping technologies, including genome-wide association studies (GWAS) and DArTseq genotyping, have further improved the identification of genomic regions associated with stress adaptation. These approaches represent an important transition from anonymous markers toward functional markers directly associated with stress-responsive genes. In that sense, Wirojsirasak et al. (2023) screened 649 candidate genes in a diversity panel evaluated under water-stressed and non-stressed conditions across plant cane and ratoon cycles. They identified 62 marker-trait associations (MTAs) in 58 genes under water-stressed, specifically associated with drought tolerance indices, explaining up to 30.52% of phenotypic variance. Associated genes were enriched in hormone signaling, transcriptional regulation, and sugar metabolism, overlapping with the antioxidant, osmotic adjustment, and hormone-signaling candidates identified in transcriptomic studies (Khueychai et al., 2015; Tang et al., 2023; Wang et al., 2025b) and reinforcing these pathways as priority targets for functional marker development. More recently, Rungmekarat et al. (2026) demonstrated the potential of DArT-seq markers to discriminate tolerant and sensitive sugarcane clones, without resolving individual candidate loci. Together, these studies converge on a still-fragmented set of drought-responsive candidates that could anchor a functional marker panel, though a formal meta-analysis or consensus QTL map across these datasets remains lacking. Because individual MTAs each explain only a modest share of phenotypic variance and drought tolerance is controlled by many loci of small effect, breeding strategies that capture genome-wide marker effects jointly, rather than a handful of validated QTLs, are expected to be needed to translate this candidate-gene knowledge into selection gains. Although promising, GWAS faced limitations in identifying robust SNP-trait associations, underscoring the inherent difficulties of such analyses in complex polyploid genomes, particularly with limited population size.

**Table 4:** Molecular approaches to study drought tolerance.

Approach	Main results (reference)
TRAP primers based on candidate genes involved in the drought tolerance response (Aquaporin, Aqua; late embryogenesis abundant protein, LEA and dehydration binding factor, DBF).	TRAP were used to evaluate genetic diversity in Brazilian germplasm (Creste et al., 2010). DBF TRAP markers were also implemented to comparatively evaluate genetic diversity of brasilean and argentinean germplasm (Perera et al., 2023).
RAPD decamer primers to identify polymorphism among five genotypes.	Highly effective SCAR marker (sequence characterized amplified region) identified for assisting selection of genotypes with drought tolerance (Srivastava et al., 2012).
Specific sequence tagged SSR markers of drought-specific candidate genes (indole-3-glycerol phosphate synthase, superoxide dismutase).	The usefulness of selecting more diverse combinations for combining drought tolerance with yield and quality characters in sugarcane is highlighted (Hemaprabha and Simon, 2012).
Large-scale candidate-gene association mapping for drought tolerance and agronomic traits.	Significant marker-trait associations (MTAs) were identified in 141 candidate genes that explained 7.93–30.52% of phenotypic variation. MTAs were involved in physiological and molecular adaptation, phytohormone metabolism, and drought-inducible genes (Wirojsirasak et al., 2023).
DARTseq for molecular characterization and agro-morphological parameters for evaluation.	Genetic distinctness and considerable genetic diversity within the mutant population, whereas GWAS did not identify statistically significant associations with agronomic traits (Rungmekarat et al., 2026).

Genomic selection (GS) offers exactly this genome-wide alternative and has emerged as a promising strategy for sugarcane breeding, particularly for complex quantitative traits controlled by multiple loci (Meena et al., 2020; Hayes et al., 2021; Mahadevaiah et al., 2021; Srithawong et al., 2026). Due to the polygenic nature of drought adaptation and strong genotype × environment interactions, GS may offer advantages over marker-assisted selection based on a limited number of loci. Although GS has not yet been specifically evaluated for drought-related traits in sugarcane, previous studies have demonstrated its potential for improving selection efficiency for agronomic and industrial traits (Gouy et al., 2013; Hayes et al., 2021; Mahadevaiah et al., 2021; Yadav et al., 2021; Phiri et al., 2025) suggesting that it could also become a valuable tool for accelerating the selection of drought-tolerant genotypes.

#### 2.3.3 Biotechnological approaches

Biotechnological approaches provide opportunities to directly enhance drought tolerance by modifying stress-responsive pathways with functional genes being far more frequently employed than transcription factors (**Table 5**). While the former mainly improve specific physiological and biochemical processes, transcription factors offer the advantage of simultaneously regulating multiple downstream stress-response pathways. Expanding the use of regulatory genes, either alone or in combination with functional genes, may represent a promising strategy to achieve broader and more durable drought tolerance in future sugarcane improvement programs.

**Table 5:** Transgenesis for drought tolerance.

Gen	Methodology	Main results	Reference
Pyrroline-5-carboxylate synthase *(P5CS*) catalyzes the synthesis of pyrroline-5-carboxylate, a key precursor for the synthesis of proline and ornithine.	Calli bombardment with *P5CS* driven by a stress-inducible promoter complex.	The overall capacity of transgenic plants to tolerate water-deficit stress could be assessed by the significantly higher biomass yields 12 days after withholding water. Stress-inducible proline accumulation under water-deficit stress acts as a component of antioxidative defense systems rather than as an osmotic adjustment mediator.	Molinari et al. (2007)
*Arabidopsis* Vacuolar Pyrophosphatase 1 (*AVP1)*, a conserved type I proton pumping pyrophosphatase (H+-PPase), role in acidifying the lumen and removing cytosolic pyrophosphate (PPi), a byproduct of biosynthesis.	*AVP1* gene introduced through *Agrobacterium* in apical buds.	A longer and profuse root system was observed in transgenic plants. They were able to withstand higher NaCl salt stress as well as scarcity of water thus, showing tolerance against salinity and drought stresses.	Kumar et al. (2014)
Pea DNA helicase (*PDH45*), a DEAD-box family member protein, provides salinity stress tolerance.	Pyramided *EaDREB2* through *Agrobacterium*-mediated transformation with the PDH45 gene through particle bombardment.	Co-transformation of *EaDREB2* and *PDH45* which shows higher salinity tolerance but lower drought tolerance than *EaDREB2* alone.	Augustine et al. (2015)
Glyoxalase III catalyses the conversion of methylglyoxal into D-lactate.	Sugarcane transgenic overexpressing *EaGly III* from *Erianthus arundinaceus*	Transgenics resulted in higher relative water content, chlorophyll content, gas exchange parameters, photosynthetic efficiency, proline content and soluble sugars upon water deficit stress. Higher and stable levels of superoxide dismutase and peroxidase activities were observed along with minimal lipid peroxidation during drought stress signifying the tolerance mechanism exhibited by transgenic events. There was no significant structural change observed in the root anatomy of transgenic plants.	Mohanan et al. (2020)
Expansins facilitate cell expansion by mediating pH-dependent cell wall loosening.	Overexpression through *Agrobacterium*-mediated transformation of an *EaEXPA1* from *Erianthus arundinaceus*.	Higher relative water content, cell membrane thermostability, chlorophyll content, photosynthetic efficiency and gas-exchange parameters in transgenic plants than un-transformed control plants.	Narayan et al. (2021)
Telomeric repeat-binding factor 1 (*TERF1*), a telomeric DNA-binding protein protects chromosome ends from being recognized as DNA damage.	Embryogenic callus transformed with *Agrobacterium* harbouring the *TERF1*.	Drought tolerance through increased accumulation of osmo-protectant (proline, soluble sugars and glycine betaine), decreasing reactive oxygen species (H_2_O_2_) and malondialdehyde content. This gene might have a regulatory role in the response to drought stress in sugarcane.	Rahman et al. (2021)
G-protein-coupled receptors (*GPCRs*) are components of G-protein-mediated signaling affecting plant growth, development, and stress responses.	Transgenic events overexpressing the sugarcane *ShGPCR1*.	Transgenic lines exhibited tolerance to multiple abiotic stresses, such as drought, salinity, and cold.	Ramasamy et al. (2021)
ATP citrate lyase (*ACL*) is a major enzyme responsible for the production of acetyl-CoA in cytoplasm and plays an important role in stress response.	*SoACLA-1* was transformed into sugarcane calli by *Agrobacterium*.	Transgenic lines were more tolerant to drought as compared with the wild-type plants.	Zhu et al. (2021)
Small Ubiquitin‐Like Modifier (*SUMO*) proteases play an important role in stress responses by regulating the *SUMO*‐related post‐translational modification of proteins.	Cysteine protease (OVERLY TOLERANT TO SALT‐1; *OTS1*) from *Arabidopsis thaliana* using particle bombardment	Transformed lines displayed enhanced drought tolerance, delayed leaf senescence, high relative moisture content and higher photosynthesis rate under water deficit compared to the untransformed wild type. Besides, transformed plants were able to maintain higher stomata conductance and chlorophyll content under moderate stress compared to the control genotype.	Masoabi et al. (2023)

Transcription factors	Methodology	Main results	Reference
*DREB2A* interacts with a cis-acting DRE sequence to activate the expression of downstream genes involved in drought-, salt- and heat-stress response.	Calli bombardament for stress-inducible over-expression of *AtDREB2A.*	Up-regulation of genes involved in plant response to drought stress, increased sucrose levels and improved bud sprouting, without biomass penalty.	Reis et al. (2014)
B-Box family, integrates photomorphogenic signaling with defense responses by promoting flavonoid, sinapate and glucosinolate accumulation.	Calli bombardament with *Arabidopsis thaliana AtBBX29*.	Transgenic plants maintained higher relative water content, better protecting its photosynthetic machinery and delayed senescence under water-deficit conditions. They accumulated more proline and displayed enhanced enzymatic antioxidant activity and osmolyte capacity under drought conditions.	Mbambalala et al. (2021)
Plant Nuclear factor (*NF-Y)*, one of the major classes of transcription factors involved in plant development and abiotic stress response.	Calli bombardment with the coding sequence of *NF-Y* from *E. arundinaceus*.	Sugarcane lines had higher relative water content, chlorophyll content and photosynthetic efficiency, activity of antioxidant enzymes such as superoxide dismutase, catalase, ascorbate peroxidase, and glutathione reductase, and higher proline content, lower malondialdehyde and peroxide contents.	Chinnaswamy et al. (2024)

To date, transgenic approaches have demonstrated considerable potential for improving sugarcane resistance to biotic stresses and tolerance to abiotic stresses; however, their commercial deployment remains limited. According to the International Service for the Acquisition of Agri-biotech Applications (ISAAA) (2026) database (www.isaaa.org), genetically modified sugarcane has been approved for commercial cultivation in only three countries. Brazil became the first country to authorize commercial cultivation in 2017, initially releasing the insect-resistant varieties CTC20Bt (expressing *Cry1Ab*) and CTC9001Bt (expressing *Cry1Ac*) for the control of the sugarcane borer (*Diatraea saccharalis)*. Since then, four additional Bt varieties have been approved. In 2018, Indonesia approved the drought-tolerant event NXI-4T, developed through a collaboration between PTPN XI, Jember University, and Ajinomoto Company. This event carries the *RmBetA/betA* gene from *Rhizobium meliloti*, which encodes choline dehydrogenase, an enzyme involved in the synthesis of osmoprotective compounds, and was introduced using *Agrobacterium tumefaciens*-mediated transformation. More recently, in June 2024, Pakistan authorized the commercial release of two genetically modified sugarcane varieties developed by the University of Agriculture, Faisalabad: CABB-IRS, resistant to insect pests, and CABB-HTS, tolerant to herbicides. These examples demonstrate that transgenic sugarcane can provide practical solutions to major production constraints. Nevertheless, commercial adoption remains restricted, and regulatory approval is still pending or highly constrained in many major sugarcane-producing countries. Consequently, the widespread deployment of genetically modified sugarcane will depend not only on continued technological advances but also on regulatory frameworks, biosafety assessments, and public acceptance. Argentina provides a representative example of these challenges. Although the country has a well-established, science-based regulatory framework for genetically modified crops and is among the world’s leading adopters of agricultural biotechnology, no transgenic sugarcane variety has yet been approved for commercial cultivation. A glyphosate-tolerant sugarcane event has already received favorable assessments from the National Advisory Commission on Agricultural Biotechnology (CONABIA), the National Food Safety and Quality Service (SENASA), and the Secretariat of Agricultural Markets (Ostengo et al., 2022). However, final authorization by the Ministry of Agriculture remains pending, illustrating that commercialization of transgenic sugarcane requires additional regulatory and policy decisions beyond favorable biosafety evaluations.

## 3 Nutrition focused on Nitrogen

Sugarcane is a highly Nitrogen-demanding crop due to its rapid growth, prolonged growth cycle, and substantial biomass accumulation. Both carbon fixation and sugar production are directly affected by N deficiency in sugarcane and, unfortunately, sugarcane varieties are not highly efficient in N uptake (Robinson et al., 2007). The adoption of measures aimed at rationalizing N use and improving its efficiency has become urgent in the aforementioned context. From a sustainability perspective, Nitrogen fertilizer application helps alleviate food security challenges; however, they represent the primary source of N pollution, since N losses exacerbate climate change, leading to the degradation of soil, water, and air quality, as well as biodiversity decline (Aslam et al., 2024). It must be highlighted that up to 60% of the N fertilizer applied in sugarcane systems can be lost through nitrate leaching, ammonia volatilization, and denitrification processes, including the release of the potent greenhouse gas N_2_O (Robinson et al., 2007). For instance, in Brazil approximately 60% of N-based fertilizer applied to sugarcane fields is recovered by plants and soils, whereas N losses to leaching and N_2_O emissions can average 5.6 and 1.84% of the total applied N, respectively (Otto et al., 2016). Given the environmental consequences associated with N losses and the relatively low NUE of sugarcane, improving N management has become a central component of sustainable production systems.

Drought events associated with climate change underscore the importance of Nitrogen nutrition not only as a determinant of crop productivity but also as a component of sugarcane adaptation to environments with limited water availability (Abhiram et al., 2025). Drought restricts water movement in the soil, thereby reducing Nitrogen transport to the roots via mass flow; this limits Nitrogen uptake and lowers NUE in the plant (Kulrat et al., 2025). Consequently, reduced Nitrogen uptake and assimilation lead to a progressive decline in leaf Nitrogen content, given that 60% of leaf N in C4 plants is located within the chloroplasts (Mengesha, 2021). Water deficit induces thylakoid membrane degradation, compromising chloroplast integrity and the efficiency of the photosynthetic apparatus, which is responsible for carbon assimilation and the energy supply required for Nitrogen metabolism (Monteoliva et al., 2021). In a study of sugarcane genotypes grown under early-season drought conditions, Kulrat et al. (2025) found a strong positive correlation between photosynthetic Nitrogen use efficiency (PNUE) and stalk yield (r = 0.89 for plant cane, and r = 0.92 for ratoon cane). This indicates that maintaining PNUE under water deficit is vital for sugarcane productivity. Furthermore, Bascuñán-Godoy et al. (2018) demonstrated that Nitrogen contributes to photoprotective mechanisms during drought-induced senescence by enhancing the dissipation of excess light energy, thereby reducing the oxidative damage associated with photosynthetic limitations under water deficit. Thus, N can also play a role in protecting the plant under drought stress conditions. Therefore, improving NUE should be considered an important strategy for enhancing sugarcane resilience in the face of future climate scenarios (Abhiram et al., 2025).

In light of climate change and the relatively low efficiency of N use, the future of sugarcane production depends on consolidating sustainable production systems based on circular economy principles. As a major bioenergy crop, sugarcane offers unique opportunities to integrate renewable energy production with the recycling of agricultural and industrial residues, thereby improving resource use efficiency (Aguilar-Rivera, 2022; Jimenez et al., 2025). In this context, integrating efficient N management, technological innovation and high-NUE genotypes will be essential to maximize productivity while minimizing environmental impacts.

### 3.1 Strategies for sustainable Nitrogen management

Improving NUE in sugarcane requires the integration of complementary management strategies that optimize fertilizer use while enhancing biological processes involved in Nitrogen acquisition and recycling. Among these, best management practices, biological interactions in the rhizosphere, and the development of high-NUE cultivars represent the main pillars of sustainable Nitrogen management.

#### 3.1.1 Agronomic and biological management strategies

Traditional approaches to improving NUE have primarily focused on optimizing fertilizer management. The implementation of the 4R nutrient stewardship principles, applying the right fertilizer source, at the right rate, at the right time, and in the right place (Bruulsema et al., 2009), together with enhanced-efficiency fertilizers and nitrification or urease inhibitors, has substantially reduced N losses while increasing fertilizer recovery (Abhiram et al., 2025). Enhanced-efficiency fertilizers include slow- or controlled-release formulations (CRFs), such as polymer-coated urea (PCU), nutrient-coated urea, and bioactive bacterium-coated urea (Castro et al., 2022). Compared with conventional urea, CRFs reduce ammonia (NH_3_) and nitrous oxide (N_2_O) emissions by 32.7% and 25.0%, respectively (Lu et al., 2023), while also improving crop performance, with reported increases of up to 50% in biomass production and approximately 7% in sugar yield (Rathnappriya et al., 2022; Swify et al., 2024). Similarly, nitrification and urease inhibitors can reduce N volatilization losses by 50–60% and greenhouse gas emissions by 40–95%, while increasing sugarcane productivity by up to 31% (Fan et al., 2022; Aslam et al., 2024).

Beyond improving fertilizer management and biological nutrient acquisition, sustainable Nitrogen management also relies on strategies that promote nutrient recycling and increase the efficiency of fertilizer delivery. In this context, recycling agro-industrial by-products, particularly the application of vinasse combined with organomineral fertilizers, has been associated with yield increases of 16–24%, improved soil conditions, and reduced dependence on synthetic fertilizers (Carpanez et al., 2022; Montiel-Rosales et al., 2025). Besides reducing N volatilization losses and improving NUE, vinasse valorization also contributes to circular production systems through biogas generation (Jimenez et al., 2025). Likewise, nanotechnology has emerged as a promising approach to improve fertilizer delivery and nutrient use efficiency, with nanomaterials showing potential to increase crop productivity while reducing fertilizer inputs (Aslam et al., 2024). Nevertheless, further studies are still required to evaluate the potential accumulation of nanoparticles in sugarcane production systems. Collectively, these complementary approaches illustrate how sustainable Nitrogen management is progressively shifting from maximizing fertilizer inputs toward optimizing nutrient cycling, resource use efficiency, and environmental performance within circular production systems.

Moreover, precision agriculture in sugarcane enables the monitoring and management of crop performance under specific environmental conditions, optimizing management resources such as water, fertilizers, and pesticides and promoting sustainability. Key components are modern remote sensing technologies, such as drones and satellite imagery, which enable real-time monitoring of soil moisture, nutrient levels, and plant health (Riaz Ud Dean, 2025). Recently, a study developed a time- and cost-effective, technically acceptable remote sensing method to estimate the spatial variability of sugarcane nutrients. Results revealed a strong spatial relationship among soil moisture, nutrient status, and plant health, demonstrating its value as a precision agriculture tool for optimizing nutrient management in sugarcane (Picado et al., 2025). Integrating intercropping systems—particularly involving sugarcane and legumes—emerges as a key strategy to enhance NUE and sustainable productivity, while mitigating the impacts associated with intensive monoculture and the excessive use of Nitrogen fertilizers. This cropping system promotes plant nutrition and growth by selectively recruiting a consortium of growth- and stress-regulating bacteria that facilitate biological Nitrogen fixation (BNF) and increase nutrient availability in the rhizosphere (Fallah et al., 2026; Pang et al., 2026), while simultaneously could improve crop resilience to stresses such as drought. Since legumes obtain Nitrogen primarily through atmospheric N_2_ fixation, whereas sugarcane relies predominantly on mineral Nitrogen available in the soil, this complementarity reduces direct competition for the nutrient; it also contributes to enriching soil Nitrogen stocks and microbial biomass, as well as improving nutrient cycling efficiency (Liu et al., 2021). In sugarcane production systems, the use of legumes in intercropping or rotation has been linked to yield increases of 27–43% and biological Nitrogen inputs of 41–71 kg N ha^−1^ (Abhiram et al., 2025). Studies demonstrate that sugarcane-peanut intercropping significantly boosts land-use efficiency, with the Land Equivalent Ratio (LER) ranging from 1.21 to 1.42, and increases alkali-hydrolyzable soil Nitrogen compared to monoculture (Fallah et al., 2026). This approach can also help mitigate the effects of acidification induced by the excessive use of Nitrogen fertilizers, a process that reduces N availability in the rhizosphere and impairs microbial communities involved in Nitrogen fixation and the mobilization of essential nutrients (Pang et al., 2026). By fostering microbial diversity and functionality in the rhizosphere, the consortium can enhance nutrient cycling and increase the stability of the production system under adverse environmental conditions. Similar to the genotype-dependent responses described for drought-associated rhizosphere interactions, the benefits of intercropping also depend on the ability of individual sugarcane cultivars to establish distinct microbial communities and recruit Nitrogen-fixing bacteria under consortium conditions (Liu et al., 2021).

#### 3.1.2 Genotype-dependent interactions with beneficial microorganisms

While agronomic practices can create favorable conditions for BNF, the extent to which sugarcane benefits from microbial associations depends largely on the compatibility between plant genotype and beneficial microorganisms. Diazotrophic bacteria associated with sugarcane, including species from the genera *Bacillus*, *Pantoea*, and *Bradyrhizobium*, have been shown to harbor Nitrogen fixation-related genes (such as *nifH*) and contribute to plant growth and Nitrogen acquisition (Matos et al., 2021; Singh et al., 2020, 2021). In Brazil, BNF represents an important component of sugarcane Nitrogen nutrition, with estimated contributions ranging from 18 to 57% of crop N demand depending on genotype and environmental conditions (Silvestre et al., 2026), highlighting its potential to partially reduce reliance on synthetic Nitrogen fertilizers.

Beyond atmospheric Nitrogen fixation, diazotrophic microorganisms promote plant growth through multiple complementary mechanisms, including phytohormone production (e.g., indole-3-acetic acid), stimulation of root development, and modulation of Nitrogen assimilation via enzymes such as nitrate reductase and glutamine synthetase (Carvalho et al., 2022; Silva et al., 2025). Interestingly, endophytic Nitrogen-fixing bacterium also enhances sugarcane performance under drought stress (Nong et al., 2023). The magnitude of benefits varies substantially among sugarcane genotypes, indicating that plant genetic background strongly influences the establishment and efficiency of beneficial plant–microbe interactions. Responsive genotypes, such as SP70–1143 and RB867515, exhibit molecular characteristics associated with enhanced bacterial association, including reduced expression of defense-related genes such as NBS-LRR receptors, suggesting a more permissive environment for beneficial microbial colonization (Carvalho et al., 2022). Conversely, genotypes such as Chunee (*S. barberi*) and IACSP95–5000 display lower BNF contributions (approximately 32%), which have been associated with differences in auxin signaling, microbial recognition, and Nitrogen metabolism regulation (Carvalho et al., 2022).

The effectiveness of microbial inoculation is further influenced by environmental conditions, particularly soil Nitrogen availability. Responses to inoculants are generally greater under low-fertility conditions or reduced Nitrogen supply, where plants may rely more strongly on biological inputs to maintain growth (Oliveira et al., 2006). Recent studies within the *Saccharum* complex indicate that wild relatives, such as *S. spontaneum* genotype US72-1319, can exhibit exceptional responsiveness to microbial inoculation, with biomass increases of up to 253%, suggesting that ancestral germplasm may harbor valuable traits for improving NUE (Silva et al., 2025). Therefore, future breeding strategies should consider plant–microbe compatibility as an additional selection criterion, integrating genotype, microbial strain, and environmental interactions to optimize biological Nitrogen acquisition in sugarcane.

### 3.2 Conventional breeding

The development of new sugarcane varieties also represents an effective strategy to enhance NUE, defined as the plant’s ability to absorb Nitrogen from the soil and assimilate it internally, converting the nutrient into biomass (Leal Junior et al., 2025). NUE represents a complex genetic trait influenced by environmental factors, such as temperature, precipitation, and radiation, as well as by biological processes including Nitrogen uptake, assimilation, and remobilization (Govindasamy et al., 2023). NUE can be quantified by two main indices: Nitrogen uptake efficiency (NUpE) and internal Nitrogen utilization efficiency (NUtE, also known as iNUE) (Leal Junior et al., 2025). NUpE is defined as the ability of plants to absorb N from the soil, and NUtE as the ability to utilize the absorbed N to produce biomass or grains (Lebedev et al., 2021). In a previous study, a subset of 61 randomly selected genotypes derived from a cross between a *S. officinarum* clone and an Australian commercial cultivar was grown in the glasshouse under limiting (low) and non-limiting (high) N supply. Several genotypes showed high iNUE at both N levels, suggesting that the considerable genetic variation for iNUE in sugarcane can be exploited for breeding (Robinson et al., 2007). Accordingly, considerable genetic variation in Nitrogen uptake and utilization has been documented among sugarcane cultivars under both low and high Nitrogen supply, highlighting the breeding potential for improving NUE (Yang et al., 2019; Zeng et al., 2020; **Table 6**).

**Table 6:** Sugarcane genotypes from the main producing countries and their classification regarding nitrogen use efficiency (NUE).

Country	N fertilizer dose recommendations (kg N/ha)			Genotype	NUE	Reference
	Plant crop	Ratoon crop	Reference			
Brazil	60 - 90	80 - 160	Quaggio et al. (2022)	RB92579	High	Leal Junior et al. (2025)
				RB966928	High	Leal Junior et al. (2025)
				RB036152	High	Leal Junior et al. (2025)
				RB72454	Medium	Leal Junior et al. (2025)
				CTC4	High	Leal Junior et al. (2025)
				SP81-3250	High	Leal Junior et al. (2025)
				RB855536	Low	Leal Junior et al. (2025)
				RB867515	Medium	Leal Junior et al. (2025)
India	150 - 250	200 - 400	Robinson et al. (2011), Shukla et al. (2017)	Co 95020	High	Mohanraj et al. (2020)
				Co 07004	High	Mohanraj et al. (2020)
				Co94012	Medium	Mohanraj et al. (2020)
				Co07017	Low	Mohanraj et al. (2020)
China	500 - 700	500 -700	Robinson et al. (2011), Yang et al. (2024)	ROC22	High	Yang et al. (2019), Zeng et al. (2020)
				GT32	High	Yang et al. (2019)
				YT94-128	High	Yang et al. (2019)
				NCo376	Low	Yang et al. (2019)
				GT42	Medium	Yang et al. (2019)
				GXASF180-1-11	High	Anas et al. (2021a, 2021b)
				GT11	Low	Anas et al. (2021a, 2021b), Zeng et al. (2020)
				Badila	Low	Yang et al. (2019)
				LC05-136	High	Anas et al. (2021b)
Australia	120 - 160	140 - 180	Robinson et al. (2011), Bell and Moody (2014)	Q165	High	Anas et al. (2021b)
				Q177	Low	Anas et al. (2021b)

Substantial differences in NUE have been reported among commercial sugarcane cultivars (**Table 4**). For example, Brazilian cultivars have shown high NUE across different production environments, whereas in China the cultivar ROC22 has been widely recognized for its high biomass production and tolerance to low Nitrogen availability (Yang et al., 2019; Zeng et al., 2020; Yuan et al., 2025).

These findings support the incorporation of NUE components (NUpE and NUtE) as selection criteria in breeding programs to reduce dependence on Nitrogen fertilizers while maintaining crop productivity and promoting more sustainable production systems (Leal Junior et al., 2025; Anas et al., 2021a).

In several sugarcane breeding programs, varieties are typically selected under growth conditions that reflect high N fertilization regimes, and these practices may have inadvertently led to the loss of traits associated with improved NUE (Robinson et al., 2007). Nevertheless, indirect genetic gains for NUE have been reported; Over a 70-year period, NUE and water use efficiency (WUE) showed a consistent linear increase with the year of release of sugarcane varieties from an Argentine breeding program (Acreche, 2017). These findings suggest that conventional breeding has the potential to improve NUE, while also highlighting the importance of evaluating genotypes under contrasting Nitrogen conditions to facilitate the identification of superior performers across environments (Robinson et al., 2007). In addition to optimizing selection within elite germplasm, conventional breeding can also benefit from broadening the genetic base available for selection. Because biological Nitrogen fixation contributes substantially to Nitrogen acquisition in sugarcane, expanding genetic variation for BNF represents a complementary strategy for improving NUE. Accordingly, conventional breeding can also benefit from broadening the genetic base available for selection. In this context, Luo et al. (2023) provided the first evidence of extensive genetic variation in BNF capacity within the sugarcane progenitor *S. spontaneum*. Molecular characterization of high-BNF accessions unraveled the diversity of gene activity and metabolic pathways associated with carbon, Nitrogen, amino acid, and hormone metabolism, suggesting that distinct metabolic strategies underlie Nitrogen-related processes. The extensive variation in BNF capacity identified in *S. spontaneum* highlights its value as a genetic resource for broadening the breeding pool and incorporating traits associated with enhanced biological Nitrogen fixation and Nitrogen acquisition into elite sugarcane germplasm. These findings also suggest that expanding the genetic diversity available to breeding programs may facilitate future improvement of NUE, a trait whose complex genetic architecture makes genetic improvement particularly challenging. Beyond broadening the genetic diversity available for selection, future sugarcane breeding programs should also consider the capacity of genotypes to establish efficient interactions with beneficial microorganisms and to respond consistently to microbial inoculants. Increasing evidence indicates that sugarcane genotypes differ in their ability to recruit and benefit from beneficial microbial communities, suggesting that plant–microbiome interactions represent an additional source of exploitable genetic variation for sustainable crop improvement. Integrating these interactions into breeding programs could enhance Nitrogen acquisition, improve resource-use efficiency, and increase resilience under low-input production systems (Hanif et al., 2024).

### 3.3 Breeding innovations

The identification of substantial genetic variation for Nitrogen uptake, utilization efficiency, and BNF demonstrates that NUE is a promising target for sugarcane breeding. Nevertheless, the complex genetic architecture of the trait, together with its strong environmental influence and the highly polyploid nature of the sugarcane genome, also limits the efficiency of conventional selection. As a result, breeding innovations integrating molecular markers, genomics, transcriptomics, genome editing, and beneficial plant–microbe interactions are becoming increasingly important to accelerate the development of sugarcane cultivars with improved NUE.

#### 3.3.1 Candidate gene identification: transcriptomic and proteomic studies

At the molecular level, a few approaches (**Table 3**) have been used to investigate the genetic basis of NUE. These studies often focus on mechanisms and multiple determinants without considering the physiological processes that directly influence plant productivity (Pathak et al., 2011). Sugarcane genotypes with different NUE in the field differed in expression of glutamine synthetase (*GS*), the central enzyme for N metabolism in plants. Five genes encoding cytosolic (*scGS1.a, scGS1.b* and *scGS1.c*), plastidic (*scGS2*), and dodecameric (*scGSI*) GS were identified. The study revealed that scGS1.a and scGS1.b can be important for N assimilation in sugarcane, not only for the N provided by BNF, but also for that supplied by the soil and through remobilization, explaining, at least in part, the variation in the performance of sugarcane genotypes under low N input (de Matos Nogueria et al., 2005).

Another analysis of differential gene expression revealed an over-representation of different genes and pathways in two genotypes with high-BNF capacity, demonstrating that similar fixation efficiency can be achieved through distinct molecular strategies (Luo et al., 2023). The aforementioned complexity and plasticity of the regulatory networks governing plant–diazotroph interactions underscore the challenges of breeding for enhanced NUE. Interestingly, RNA-seq under symbiotic Nitrogen fixation showed enrichment of glutathione metabolism, carbon fixation, and redox-related pathways (Nong et al., 2023).

Comparative transcriptome analysis under Nitrogen-deficient conditions demonstrated that *S. spontaneum* sub-genome contributes genes involved in Nitrogen and carbohydrate metabolism, photosynthesis, and amino acid metabolism, highlighting it as a valuable genetic resource for improving NUE in modern sugarcane cultivars. Furthermore, weighted gene co-expression network analysis identified key Nitrogen-responsive genes, including the high-affinity nitrate transporter *ScNRT2*, a cell membrane-localized protein. Functional validation showed that its heterologous expression enhanced plant growth under low-nitrate conditions, while its interaction with *NAR2.1* suggested a conserved regulatory module involved in Nitrogen uptake and adaptation to Nitrogen deficiency (Hui et al., 2025). Another promising candidate gene supporting the breeding of high-NUE cultivars, the transcription factor *ScMYB61.1*, was identified by transcriptomic analyses of seedlings (Kiet et al., 2025).

Integrative analyses of enzyme activities, gene expression, and correlation networks indicated that high NUE performance resulted from coordinated, organ-specific enhancement of N transport and assimilation. Higher glutamine synthetase (GS) activity was confirmed together with glutamate synthase (GOGAT) activity, as well as earlier and stronger induction of the high-affinity nitrate transporters NRT2 and NRT2.2, accompanied by dynamic network reorganization under low N conditions. Interestingly, early activation of nitrate transport promotes downstream N assimilation, thereby sustaining growth under N limitation and providing potential molecular targets for breeding high-NUE sugarcane cultivars (Qin et al., 2026). Accordingly, transcriptomic and co-expression network analyses identified N metabolism and zeatin biosynthesis as key pathways involved in sugarcane responses to varying Nitrogen availability. The concentration-dependent regulation of these pathways suggests coordinated control of Nitrogen assimilation and cytokinin-related processes, providing candidate genes and regulatory networks that could be exploited in breeding programs (Wang et al., 2025).

Multi-omics frameworks also shed light on macronutrient dynamics under limiting conditions. Profiling of sugarcane roots colonized by AMF under Nitrogen starvation demonstrated that coordinated transcriptomic, proteomic, and metabolomic reprogramming regulates Nitrogen acquisition, phosphorus and potassium mobilization (Liu et al., 2026). This underscores the interconnected nature of macronutrient signaling networks, even when a single nutrient is the primary experimental focus. These findings illustrate that multi-omics frameworks can successfully resolve genotype-specific regulatory networks that link nutrient status, hormonal signaling, carbon partitioning, and stress adaptation.

#### 3.3.2 Molecular marker-based approaches: MAS

Traits related to nutrient use efficiency such as root morphology and internal remobilization are not easily visualized, turning related research laborious for a high biomass, deep rooted plant like sugarcane. Consequently, phenotyping root architecture is one of the main bottlenecks for nutrient research in sugarcane (Krishnapriya et al., 2025), limiting available data. Though nutrient efficiency QTLs are scarce, Whan et al. (2010) reported the first bi-parental QTL mapping study for NUE. Significant MTAs were identified for shoot and root biomass, shoot N accumulation, iNUE, leaf protein content, and glutamine synthetase activity, explaining individually up to 19% of variation observed. Finding also suggests that genetic variation for iNUE may be greater in ancestral or unimproved germplasm than in modern commercial cultivars, highlighting the potential value of exotic germplasm for broadening the genetic basis of NUE (Whan et al., 2010). Overall, these results provide an initial foundation for incorporating QTL associated with N-use traits into sugarcane breeding programs. These early QTL findings provide a genetic framework that can be complemented by high-density marker approaches, GWAS, and GS to dissect the polygenic basis of NUE and improve selection efficiency in sugarcane since to date the scarcity of robust molecular markers further restricts MAS (Yang et al., 2026).

#### 3.3.3 Biotechnological approaches

Approaches such as RNA interference (RNAi), transgene overexpression, and gene-editing technologies provide promising strategies to accelerate sugarcane breeding. However, to date, only limited success has been achieved in improving NUE. To date, the study by Snyman et al. (2015) represents the only reported transgenic approach specifically aimed at improving NUE in sugarcane. Embryogenic callus was bombarded with barley alanine aminotransferase cDNA (AlaAT) under rice OsAnt1 promoter. Under low-Nitrogen conditions, transgenic lines expressing the AlaAT gene exhibited enhanced performance compared with untransformed controls, demonstrating the potential of this strategy for improving NUE. Interestingly, transgenic lines differed in terms of their NUpE and NUtE contributions to the NUE, serving as valuable tools for understanding these processes.

## 4 Nutrition focused on phosphorus

Achieving high sugarcane yields depends heavily on adequate nutrient management, with phosphorus (P) being particularly critical due to its essential role in plant growth, development, and productivity. Phosphorus is the second major macronutrient required by plants and is involved in key biological processes, including cell division, photosynthesis, respiration, nucleic acid synthesis, adenosine triphosphate production, and enzyme regulation (Fernandes et al., 2023; Santos et al., 2025). In sugarcane, adequate P nutrition contributes to early root establishment, sprouting, tillering, improved photosynthetic performance, sugar synthesis and accumulation, and enhanced resistance to abiotic stresses (Tarumoto et al., 2022). Conversely, P deficiency results in fewer, shorter and thinner stalks, delayed growth, reduced leaf area, impaired physiological responses, decreased ratoon vitality, and lower juice quality (Santos et al., 2025).

Climate change is expected to intensify challenges associated with P nutrition by altering soil processes, plant development, and stress responses. Sugarcane phenological stages depend on optimal temperature, nutrient availability, and soil moisture conditions; when these conditions are disrupted during germination, bud dormancy, poor seedling establishment, and increased mortality may occur (Verma et al., 2022).

### 4.1 Strategies for sustainable phosphorous management

Phosphorus management represents a major challenge for sustainable sugarcane production because many high-yielding regions rely on highly weathered soils with strong P-fixing capacity and intrinsically low P availability. Although P is abundant in soil, it is frequently present in forms that cannot be directly absorbed by plants, resulting in low nutrient availability (Wu et al., 2022). In soil, P is involved in several reversible processes, including immobilization in non-living organic matter and microbial biomass, adsorption to colloidal particles, and precipitation as calcium, aluminum, or iron phosphates (Fernandes et al., 2023). Consequently, P must be mineralized, desorbed, or dissolved into the soil solution before it can be efficiently taken up by crops (Wu et al., 2022). Due to these interactions, P has the lowest use efficiency among major macronutrients in agricultural systems (Santos et al., 2025).

The high dependence on external P inputs has raised concerns regarding both fertilizer efficiency and long-term resource sustainability. The 5R global P sustainability framework proposed by Withers et al. (2015) emphasizes five complementary strategies: Realigning P inputs with crop demand, Reducing P losses to water bodies, Recycling existing bioresources more efficiently, Recovering and reusing P from waste streams where feasible, and Redesigning production systems to enhance long-term sustainability (Soltangheisi et al., 2019). In sugarcane, P fertilization practices vary according to soil characteristics and expected productivity. In Brazil, recommended P inputs at crop establishment range from 26 to 52 kg P ha^−1^ depending on soil P availability, with approximately 13 kg P ha^−1^ applied in subsequent ratoon cycles under standard conditions (Soltangheisi et al., 2019). More broadly, recommended rates range from 160–350 kg ha^−1^ for plant cane and 0–60 kg ha^−1^ for ratoon cane in Brazil (Cantarella et al., 2022), whereas in China superphosphate applications may reach 1,500–2,250 kg ha^−1^ (Zhong et al., 2025). However, because P fixation reduces fertilizer efficiency to approximately 10–25%, excessive applications may contribute to environmental problems such as eutrophication while accelerating depletion of finite P reserves (Pang et al., 2024).

#### 4.1.1 Agronomic and biological management strategies

Given these limitations, improving P use efficiency (PUE) in sugarcane requires integrated approaches that combine improved nutrient management, biological interventions, and genetic breeding. Sugarcane genotypes exhibit substantial variability in yield response to P fertilization, suggesting that fertilizer inputs could be optimized through the selection of cultivars better adapted to specific soil environments. In sugarcane, PUE has been associated with increased root biomass and greater root surface area, which enhance soil exploration and nutrient acquisition (Arruda et al., 2016). Therefore, the development of P-efficient cultivars represents a promising strategy to reduce fertilizer requirements while maintaining productivity.

Biological approaches based on beneficial microorganisms have emerged as potential tools to improve P availability in low-P environments. Arbuscular mycorrhizal fungi (AMF) facilitate plant acquisition of water and mineral nutrients through a mutualistic association in which fungi receive carbohydrates from the host plant (Moura et al., 2024). In sugarcane and other crops, mycorrhizal P uptake occurs through both root and hyphal pathways, as fungal hyphae extend beyond the root depletion zone, increasing the explored soil volume and accessing smaller soil pores than roots alone (Pang et al., 2024). Furthermore, AMF hyphae release amino acids, carbohydrates, hormones, acid phosphatases, and organic acids, which contribute to P mobilization by reducing soil pH and promoting phosphate conversion into available forms (Pang et al., 2024). However, AMF-based strategies are generally more effective under moderate nutrient limitation and may have reduced performance in highly depleted soils. Therefore, their effectiveness depends on environmental conditions and biological interactions. Accordingly, the sensitivity of the AMF communities significantly differed between genotypes under high soil acidity and P pollution (Qin et al., 2025).

Complementary to AMF, phosphate-solubilizing microorganisms (PSM) represent another biological strategy to enhance P availability and utilization efficiency. PSM include diverse bacterial groups, including *Bacillus*, *Pseudomonas*, and *Rhizobium*, among other recognized genera with potential roles in P mobilization (Fernandes et al., 2025). In sugarcane systems, PSM have shown potential to improve P acquisition under P-deficient conditions by converting unavailable P forms into more accessible compounds (Soltangheisi et al., 2019; Santos et al., 2025). Through secretion or organic matter decomposition, PSM produce low-molecular-weight organic acids that dissolve inorganic phosphates and chelate Ca, Al, and Fe ions, increasing soluble phosphate availability in the rhizosphere (Santos et al., 2025). Additionally, PSMs produce hydrolytic enzymes such as phosphatases and phytases, which contribute to organic P mineralization through dephosphorylation processes and the release of orthophosphate from phytate, respectively (Silva et al., 2026). Juntahum et al. (2025) found that applying a microbial consort of PSB and AMF with filter cake compost resulted in higher yield, microbial activity and soluble P content, even when compared to mineral fertilization. Nevertheless, microbial effectiveness depends on soil properties, microbial persistence, and plant–microbe interactions, highlighting the need for long-term studies under commercial sugarcane conditions (Soltangheisi et al., 2019). Additionally, environmental stress disrupts rhizosphere function by compromising the interactions between root metabolites and the rhizosphere microbiome, which justifies the need for sustainable management in sugarcane productive systems.

### 4.2 Conventional breeding

Developing sugarcane varieties with enhanced PUE therefore constitutes an emerging breeding objective. However, compared with other major crops, the genetic basis of PUE remains poorly characterized in sugarcane although physiological variation among genotypes with contrasting PUE has been documented. Differential responses to P deficiency have been associated with root architecture, with higher PUE genotypes displaying shallower and denser root systems that promote topsoil exploration (Tarumoto et al., 2022; Yi et al., 2022). Greater shoot P uptake, leaf P concentration, and photosynthate production have also been proposed as traits associated with improved PUE and adaptation to P-limited soils (Zambrosi et al., 2014). In that sense, genetic strategies targeting root architecture, P transport, and PUE offer complementary opportunities for developing more efficient cultivars, while genomic, biotechnological, and multi-omics approaches may accelerate progress.

### 4.3 Breeding innovations

Recent advances in genomics, molecular breeding, and genome-editing technologies may provide new opportunities to accelerate the improvement of PUE in sugarcane. Although studies specifically targeting P acquisition and utilization in sugarcane remain scarce, highlighting an important opportunity for future research.

Omics approaches are also contributing to a deeper understanding of sugarcane responses to nutrient limitations and environmental constraints. Although omics studies specifically addressing PUE in sugarcane remain limited, these approaches provide valuable frameworks for dissecting the complex regulatory networks that may underlie phosphorus acquisition, utilization, and adaptation to nutrient-limited environments.

Phosphate transporters (PHTs) play central roles in P acquisition and translocation from soil into plant tissues. In sugarcane, twenty-three *ShPHT* genes have been identified by Genome-Wide in Silico and classified into five groups (*ShPHT1* to *ShPHT4* and *ShPHO1*), with proposed functions in phosphate transport, phosphate starvation responses, stress adaptation, and symporter activity (Murugan et al., 2022). These transport systems constitute attractive targets for breeding and biotechnological strategies aimed at improving PUE. However, the lack of QTL mapping populations specifically designed to investigate PUE-related traits represents an important research gap and an opportunity for future studies.

While individual omics datasets provide valuable information, multi-omics integration offers greater potential to resolve the complex regulatory networks controlling nutrient acquisition, stress responses, and carbon partitioning (Grandis et al., 2023). Recent studies suggest that tillering involves a balance between productivity and stress adaptation, indicating possible trade-offs between growth and defense responses. Interestingly, high-tillering varieties may exhibit enhanced nutrient uptake, particularly under phosphorus-limited conditions, and may use soil nutrients more efficiently through interactions with rhizosphere microorganisms (Chen et al., 2026).

It must be highlighted that despite the analytical capacity and the evident agronomic relevance of the trait, comprehensive multi-omics approaches have rarely been directed specifically toward dissecting Phosphorus acquisition and use efficiency in sugarcane, representing a critical gap for future research. Accordingly, the absence of transgenic approaches for PUE improvement may, at least in part, reflect the limited characterization of the genetic and molecular basis of PUE in sugarcane, which currently restricts the identification and validation of suitable targets for genetic engineering.

## 5 Future prospects

Genomic selection (GS) may offer advantages over marker-assisted selection based on a limited number of loci due to the polygenic nature of drought adaptation and strong genotype × environment interactions Although GS has not yet been specifically evaluated for drought-related traits in sugarcane, previous studies have demonstrated its potential for improving selection efficiency for agronomic and industrial traits (Gouy et al., 2013; Hayes et al., 2021; Mahadevaiah et al., 2021; Yadav et al., 2021; Phiri et al., 2025).

Additionally, the integration of GS with enviromics and artificial intelligence (AI) may further enhance the efficiency of breeding programs targeting climate adaptation. Combining genomic information with environmental variables, crop physiology, and high-throughput phenotyping data can improve the characterization of genotype × environment interactions and increase predictive accuracy (Varshney et al., 2021; Resende et al., 2021). AI and machine learning approaches can facilitate the analysis of complex multi-omics datasets and support decision-making in breeding pipelines (Crossa et al., 2019). In sugarcane, where drought stresses are a major constraint affecting yield stability and where genotype × environment interactions are particularly complex due to the crop’s high heterozygosity and polyploid genome, these integrative approaches are especially relevant. Emerging frameworks that couple genomic prediction, enviromics, and mechanistic crop modeling within digital twin environments may enable the simulation of sugarcane performance under water-limited conditions, including scenarios not yet observed in field trials (Wang et al., 2024). This would allow the in silico exploration of genotype × environment × management (G×E×M) interactions under current and future drought stress regimes, supporting the identification of genotypes with stable performance across contrasting water availability environments. Together, these technologies may accelerate the development of resilient sugarcane cultivars capable of maintaining productivity under increasingly variable and water-scarce environments.

Reported prediction accuracies in sugarcane vary among studies, reflecting differences in training population size, marker density, trait architecture, and prediction models. Early work based on relatively small populations (167 clones genotyped with 1,499 DArT markers) reported accuracies ranging from 0.11 to 0.62 across agronomic traits (Gouy et al., 2013), indicating that GS performance can be highly trait-dependent and sensitive to the size and composition of the training population. More recent studies based on a larger reference population of 3,984 clones genotyped with 26K SNPs have generally provided more consistent predictions. Hayes et al. (2021), using a single-step model integrating pedigree and marker information, reported accuracies of 0.30–0.44 for cane yield, sugar content, and fiber, and showed that GS could potentially increase the rate of genetic gain relative to conventional phenotypic selection. Yadav et al. (2021), using the same reference population, further demonstrated that non-additive effects may account for a substantial proportion of the genetic variance in cane yield, with extended GBLUP models incorporating dominance effects increasing prediction accuracy by at least 17% for this trait, although no improvement was observed for sugar content or fiber. Collectively, these studies demonstrate that GS accuracy in sugarcane is not determined by training population size alone, but also depends on trait architecture and model specification. Nevertheless, the results obtained with large reference populations support the value of investing in adequately sized, representative training populations, particularly when targeting complex traits. For drought tolerance, NUE, and PUE, where environmental interactions and phenotypic plasticity are expected to contribute substantially to trait variation, dedicated training populations with multi-environment phenotyping under contrasting water and nutrient regimes will likely be required to establish the accuracy and practical value of GS in sugarcane. In particular, incorporating contrasting N and P availability into training populations could enable the identification of genotypes with improved nutrient acquisition and use efficiency, while also providing an opportunity to investigate the genetic relationships among drought tolerance, NUE, and PUE.

Furthermore, the polyploid, highly heterozygous, and aneuploid structure of the sugarcane genome (Healey et al., 2024), arising from interspecific hybridization between *S. officinarum* and *S. spontaneum*, complicates both variant calling and prediction modeling, since diploid genotype classes cannot capture the full range of allele dosages at a given locus. Genomic prediction models that explicitly incorporate allele dosage, rather than diploidizing genotype calls, better represent additive and digenic dominance effects; however, Batista et al. (2021) found that in populations with a low frequency of intermediate heterozygous dosage classes, as in existing sugarcane panels, the practical gain from full dosage modeling over simpler pseudo-diploid parameterizations (as used by Yadav et al., 2021) was limited, indicating that the value of dosage-aware models depends on the diversity and ploidy structure of the specific training population. Chromosome-scale, haplotype-resolved assemblies and pangenome graphs that represent the mixed-ploidy structure of Saccharum directly are beginning to improve variant calling and dosage estimation, which should in turn sharpen both GWAS resolution and GS marker sets (Jaimes et al., 2024; Huang et al., 2026; Wang et al., 2026). Sequencing-based dosage estimation, which better captures rare and structurally variable alleles than array-based genotyping, is likely necessary to translate these genomic gains into drought-tolerance and efficient nutrient uptake breeding specifically. Resolving this genome-level bottleneck is a precondition for GS to reach its full potential in sugarcane, but on its own it does not address the second major gap identified above, the lack of a consensus set of candidate loci to prioritize within genome-wide marker sets.

Multi-omics integration offers a complementary route to close this gap, linking genome-wide variation directly to the regulatory and metabolic processes that GWAS and candidate-gene studies have so far only partially resolved for drought tolerance, while also clarifying the molecular basis of carbon partitioning and sucrose accumulation, a trait tightly linked to yield stability under stress. Proteomic profiling of contrasting high- and low-sucrose genotypes identified 507 differentially abundant proteins, 49 of which map to sugar metabolism pathways, implicating glycolytic and fermentation enzymes as key regulators of carbon flux during culm maturation (Li et al., 2022). Aono et al. (2025) integrated transcriptomic, proteomic, and metabolomic datasets across multiple genotypes using linear mixed models, machine learning, and gene co-expression networks, identifying a coordinated regulatory network underlying efficient carbon allocation to sucrose. Similarly, multi-omics analyses have shown that differential carbon allocation contributes to variation in sucrose accumulation among contrasting sugarcane genotypes (Li et al., 2025). These studies show that multi-omics approaches can resolve genotype-specific carbon allocation strategies not visible from genomic data alone. The integration of GS with enviromics and AI may further enhance the efficiency of breeding programs targeting climate adaptation: AI extending this integrative logic from sucrose accumulation to water-limited conditions specifically, and feeding the resulting priors back into genome-wide marker sets, is a natural next step for linking sucrose stability with drought resilience and for narrowing the fragmented candidate-gene landscape described above (Crossa et al., 2019; Varshney et al., 2021; Resende et al., 2021).

In sugarcane, where drought stresses along with suboptimal N and P availability are major constraints affecting yield stability and where genotype × environment interactions are particularly complex due to the crop’s high heterozygosity and polyploid genome, these integrative approaches are especially relevant precisely because the genomic and phenotypic resources needed to train specific GS models remain limited, as noted above. Emerging frameworks that couple genomic prediction, enviromics, and mechanistic crop modeling within digital twin environments may enable the simulation of sugarcane performance under water-limited conditions, including scenarios not yet observed in field trials (Wang et al., 2024). This would allow the in silico exploration of genotype × environment × management (G×E×M) interactions under current and future stress regimes, potentially overcoming the difficulty of establishing large loco training populations under contrasting field conditions, and supporting the identification of genotypes with stable performance across contrasting environments. Together, these technologies may accelerate the development of resilient sugarcane cultivars capable of maintaining productivity under increasingly variable environments.

Approaches such as transgene overexpression or gene editing through RNA interference (RNAi) and CRISPR/Cas9, provide promising strategies to accelerate sugarcane breeding despite the genomic complexity of sugarcane and the regulatory constraints (Mall et al., 2022; Srithawong et al., 2026). All of these have effectively targeted genes associated with key traits, including sucrose accumulation, lignin biosynthesis, lipid accumulation in biomass, and stress tolerance (Brant et al., 2025). In this regard, greater advances have been made toward improving drought tolerance than NUE or PUE. Although only one drought-tolerant transgenic event is currently available commercially, these achievements provide proof of concept and open new opportunities for the development of improved cultivars using emerging breeding technologies. Therefore, genomic sequencing and gene-editing techniques are innovative and essential tools for increasing productivity while minimizing environmental impacts and enhancing resilience to climate change. The development of new genotypes requires detailed knowledge of the specific characteristics of each variety (Da Cruz and MaChado, 2023). Accordingly, several studies have investigated the genetic, physiological, and agronomic traits of existing sugarcane cultivars to improve crop breeding strategies (Hoang et al., 2019; Yang et al., 2019; Barreto et al., 2021; Wirojsirasak et al., 2023; Kulrat et al., 2025; Leal Junior et al., 2025). Fortunately, several candidate genes underlying high NUE and PUE could serve as targets for genome editing to improve sugarcane.

Future research should prioritize integrated frameworks combining physiological, genomic, and microbiome-based strategies to develop sugarcane germplasm capable of maintaining productivity under low-N and P and climate-stressed conditions. Through coordinated advances, sugarcane breeding can evolve from fragmented approaches toward a strategically integrated, technology-driven framework, accelerating the development of more environmentally resilient production systems.
